# Pathological Proteins Are Transported by Extracellular Vesicles of Sporadic Amyotrophic Lateral Sclerosis Patients

**DOI:** 10.3389/fnins.2018.00487

**Published:** 2018-07-19

**Authors:** Daisy Sproviero, Sabrina La Salvia, Marta Giannini, Valeria Crippa, Stella Gagliardi, Stefano Bernuzzi, Luca Diamanti, Mauro Ceroni, Orietta Pansarasa, Angelo Poletti, Cristina Cereda

**Affiliations:** ^1^Genomic and Post-Genomic Center, IRCCS Mondino Foundation, Pavia, Italy; ^2^Department of Brain and Behavioral Sciences, University of Pavia, Pavia, Italy; ^3^Dipartimento di Scienze Farmacologiche e Biomolecolari (DiSFeB), Centro di Eccellenza sulle Malattie Neurodegenerative, Università degli Studi di Milano, Milan, Italy; ^4^Immunohematological and Transfusional Service and Centre of Transplantation Immunology, IRCCS Foundation San Matteo, Pavia, Italy; ^5^Division of General Neurology, IRCCS Mondino Foundation, Pavia, Italy

**Keywords:** amyotrophic lateral sclerosis, proteinopathy, extracellular vesicles, microvesicles, exosomes, SOD-1, TDP-43, FUS

## Abstract

Amyotrophic lateral sclerosis (ALS) is a progressive adult-onset neurodegenerative disease, that affects cortical, bulbar and spinal motor neurons, and it is considered a proteinopathy, in which pathological proteins (SOD1, TDP-43, and FUS) may accumulate and interfere with neuronal functions eventually leading to cell death. These proteins can be released from cells and transported in the body fluids by extracellular vesicles (EVs). EVs are spherical vesicles, which are classified mainly in microvesicles (MVs) and exosomes (EXOs) based on their biogenesis, size and surface markers. In this study we characterized MVs and EXOs isolated from plasma of sporadic ALS patients and healthy controls and determined their number, size and SOD1, TDP-43, and FUS protein composition. No variation was found in the number of EVs between ALS patients and controls. However, the mean size both for MVs and for EXOs resulted increased in ALS patients compared to controls. MVs derived from ALS patients were enriched in SOD1, TDP-43, phospho-TDP-43, and FUS proteins compared to CTRLs. SOD1 was generally more concentrated in EXOs than in MVs, while TDP-43 and FUS protein levels were slightly higher in MVs than in EXOs. We demonstrated that MVs and EXOs size were increased in ALS patients compared to controls and that MVs of ALS patients were enriched with toxic proteins compared to CTRLs. EXOs did not show any protein changes. These data may suggest that MVs can transport toxic proteins and might play a role in prion-like propagation of ALS disease.

## Introduction

Amyotrophic lateral sclerosis (ALS) is a progressive adult-onset neurodegenerative disease that primarily affects upper and lower motor neurons (Al-Chalabi et al., 2016). The disease is considered a proteinopathy, since spinal cord histology of ALS patients reveals abnormal accumulations of protein aggregates in motor neurons and neural accessory cells (Polymenidou and Cleveland, 2011). These aggregates (especially SOD1, TDP-43, FUS) originate from the accumulation of non-natively folded proteins that can propagate in a way similar to prion proteins (self-seeding), although neuronal cells are not infected with prions (Cereda et al., 2006, 2013; Diaz-Espinoza and Soto, 2010; Polymenidou and Cleveland, 2011; Pansarasa et al., 2018). This process could be mediated through release and uptake of protein aggregates or via extracellular vesicles (EVs) (Grad et al., 2014). Extracellular vesicles (EVs) have become an intriguing field in the study of neurodegenerative diseases. EVs are spherical vesicles classified mainly for size and biological functions in microvesicles (MVs) and exosomes (EXOs). MVs and EXOs are vesicles of 100–1,000 nm and 30–140 nm in diameter, respectively. Various studies have described both classes and although size is the simplest method to distinguish EXOs from MVs, there is a subpopulation of MVs which overlap in dimension with EXOs and *viceversa*. MVs are shed by budding of all cells plasma membrane and they express membrane receptors on their surface (Thery et al., 2006; Raposo and Stoorvogel, 2013; Cocucci and Meldolesi, 2015). MVs production is observed in a variety of cells in a resting state, but can be significantly elevated under various stimulations, including increased [Ca^2+^], cellular stress and immune system response, all mechanisms observed in ALS (Akers et al., 2013). EXOs instead are released by exocytosis of multivesicular bodies (MVBs), which can degradate or can fuse with the plasma membrane (Raposo and Stoorvogel, 2013).

Autophagy malfunction is often linked to EXOs secretion and it is well known that loss of basal autophagy, as a degradative pathway of proteins, is a cause of neurodegeneration of ALS (Baixauli et al., 2014). In ALS EVs are described in the release and uptake of misfolded/aggregated proteins like SOD1, TDP-43 and FUS and most of the evidence in the literature regards EXOs from cell cultures, but not MVs. The loading mechanism of EXOs is controlled by different pathways, like the endosomal sorting complexes (ESCRT) machinery, required for the sorting of ubiquitinated membrane proteins (Moreno-Gonzalo et al., 2014). It was observed that the presence and the propagation of mutated SOD1 (mutSOD1) and wild type SOD1 (WTSOD1) from an *in vitro* motor neuron-like cell model, NSC-34, can occur via EXOs (Gomes et al., 2007; Munch and Bertolotti, 2011; Basso et al., 2013; Grad et al., 2014). EXOs release is enhanced in astrocytes expressing mutSOD1 and it has been suggested that the process may serve to eliminate protein aggregates from cells (Urushitani et al., 2008). It was demonstrated that mutSOD1 oligomers accumulate in the endoplasmic reticulum–Golgi compartments of the endocytic pathway prior to their secretion (Pinto et al., 2017). Similar to some mutSOD1 species, TDP-43 aggregates, isolated from the brains of ALS and FTLD (frontotemporal lobar dementia) patients, self-propagate in the ubiquitinated and phosphorylated form through EXOs in cultured human neuroblastoma cells (Nonaka et al., 2013; Iguchi et al., 2016) also demonstrated that exposure of Neuro2a cells to EXOs from an ALS brain, but not from a control brain, caused cytoplasmic redistribution of TDP-43, an important marker of proteinopathy. TDP-43 is transported by cerebrospinal fluid (CSF) derived EXOs of ALS patients and healthy donors and there is no differences in TDP-43 protein level between the two groups (Feneberg et al., 2014). So far, no evidence of the presence of FUS in MVs and EXOs has been reported. Primarily, in this study we have investigated whether circulating MVs and EXOs derived from plasma of sporadic ALS patients exhibit different concentrations and size compared to healthy controls. In addition, we also examined the loading difference of pathological proteins between MVs and EXOs of sporadic ALS patients and CTRLs in order to understand EVs contribution in pathological proteins clearance.

## Materials and Methods

### Subjects

ALS diagnosis was made according to the revised El Escorial Criteria (Brooks et al., 2000) at IRCCS Mondino Foundation (Pavia). Patients with concomitant comorbidity (thyroiditis, cancer, etc.) were excluded. Blood from 30 sporadic ALS patients (SALS in the text they will be called ALS patients) was collected (mean age: 71.3 ± SD 7.5). ALS individuals harboring mutations in *SOD1*, *FUS/TLS*, *TARDBP*, *C9ORF72*, and *ANG* genes were excluded. Subjects participating in the study signed an informed consent (Protocol n. 375/04 – version 07/01/2004) in accordance with the Declaration of Helsinki. See **Table 1** for demographic and clinical characteristics. Thirty sex- and age-matched healthy volunteers free from any pharmacological treatment were recruited at the Immunohematological and Transfusional Service and Centre of Transplantation Immunology IRCCS Foundation “San Matteo” (Pavia, Italy) and used as non-neurological controls (CTRLs) after signature of the informed consent in accordance with the Declaration of Helsinki. Moreover, healthy controls were not affected by any neurological or psychiatric condition, nor were taking psychoactive drugs. This study protocol from patients and controls was approved by the Ethical Committee of the IRCCS Mondino Foundation (Pavia, Italy).

**Table 1:** List of patients and clinical features.

Number	Gender	Age of onset, year	Site of onset	ALSFRSr_	Nanosight	WB
1	M	67	S	35	x	x
2	F	56	S	43	x	x
3	M	85	B	44	x	x
4	F	79	S	32		x
5	F	59	S	32		x
6	M	65	S	41	x	x
7	M	61	S	32	x	x
8	M	62	S	29	x	x
9	M	78	S	41		x
10	M	68	S	48		x
11	F	70	S	38	x	x
12	M	64	B	39		x
13	F	67	S	33	x	x
14	M	60	B	45	x	x
15	F	65	S	42	x	x
16	F	73	B	23		x
17	M	82	S	41	x	x
18	F	63	S	27	x	x
19	M	52	S	40	x	x
20	M	69	S	42		x
21	M	70	S	40	x	x
22	F	73	S	42	x	x
23	F	63	B	34		x
24	F	67	S	44	x	x
25	F	74	S	24	x	x
26	F	67	S	22	x	x
27	F	67	S	42	x	x
28	F	78	B	26	x	x
29	M	75	B	27		x
30	M	80	B	41		x


*M, male; F, female; S, spinal; B, bulbar; ALSFRS, ALS Functional Rating Scale; WB, Western Blot. Samples used for Nanosight and for Western blot are marked by an x.*

### Isolation of MVs and EXOs

**FIGURE 1:**
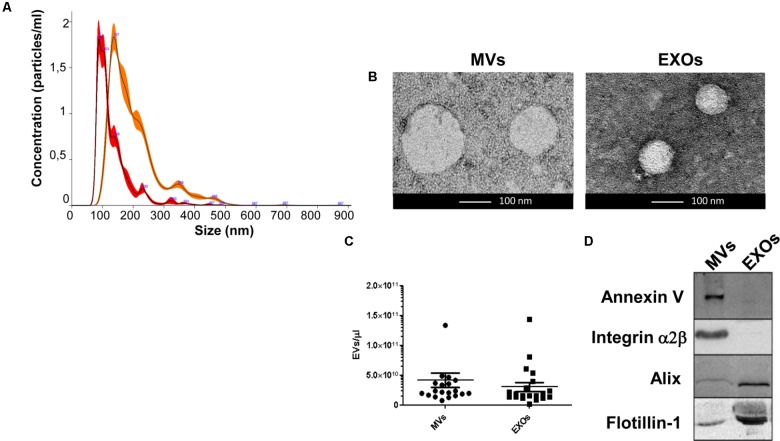
Analysis of dimension and markers of MVs and EXOs from plasma of healthy controls. **(A)** Nanoparticle distribution by NTA confirmed the purity of MVs (orange line-mode = 142.8 ± 6.0 nm) and EXOs (red line-mode = 96.7 ± 4.7 nm); **(B)** Representative images obtained by transmission electron microscopy (TEM) of MVs (two MVs of about 150 nm) and EXOs (two EXOs of about 90 nm) from plasma of a healthy control (Scale bar: 100 nm); **(C)** Representative dot plot of the concentration of plasma derived MVs and EXOs of 20 healthy individuals showed no variation between the two groups; **(D)** Western Blot of MVs and EXOs markers in MVs and EXOs samples showed the presence of Annexin V and Integrin α2β only in MVs pellet and Alix and Flotillin-1 especially in the EXOs fraction.

Venous blood (7 mL) was collected in sodium citrate tubes from all patients and controls. Within 1 h it was centrifugated at 1,000 g for 15 min to separate plasma, followed by an additional centrifugation at 1,600 g for 20 min to remove platelets. Platelet-free plasma was then transferred to a new tube and snap frozen at -80°C. Prior to the analysis, platelet-free plasma was thawed on ice and it was centrifuged at 20,000 g for 1 h with Centrifuge 5427 R (Eppendorf, Italy). The pellet was washed with 0.22 μm filtered PBS and centrifuged for 1 h at 20,000 g. The pellet was then processed for MVs analysis. The supernatant of MVs was filtered through a 0.2 μm filter and spun in an Optima MAX-TL Ultracentrifuge at 100,000 g for 1 h at 4°C. After ultracentrifugation, the supernatant was removed and the EXOs pellet was washed with 1 mL of filtered PBS at 100,000 g for 1 h at 4°C (Thery et al., 2006). The obtained EXOs pellet was processed for analysis. Western Blot analysis for MVs markers (Annexin V-Abcam, Inc., United States- and Integrin α2β Santa Cruz Biotechnology, Inc., United States) and for EXOs markers (Alix-Abcam, Inc., United States and Flotillin-BD Biosciences, United States) and Nanoparticle-tracking analysis (NTA) were run to confirm MVs and EXOs purity (**Figure 1**).

### Nanoparticle-Tracking Analysis (NTA) of MVs and EXOs

Twenty patients and twenty CTRLs were analysed by NTA using a NS300 instrument (NanoSight, Amesbury, United Kingdom) in order to detect size and concentration of MVs and EXOs. For a more accurate detection, MVs and EXOs samples were diluted with PBS to an optimal concentration (10^7^–10^9^ particles/ml). After dilution, 1 mL of diluted sample was loaded on the machine and read in a rate of about 30 frames/s. Particle movement videos (60 s/video) were recorded three times per test and size and mean concentration were analysed by the NTA software (version 2.2, NanoSight). The results of NTA were presented as the mean of the three tests.

### Transmission Electron Microscopy (TEM)

Transmission electron microscopy was used to study the morphology of MVs and EXOs. For TEM, 40 μL of vesicle suspension were placed on a carbon-coated EM grid, and 0.4 μL of 25% glutaraldehyde was added. Vesicles were then allowed to settle onto the grid overnight at 4°C. Grids were then blotted on filter paper and stained for 30 s with 2% uranyl acetate. After further blotting and drying, samples were directly observed on a Tecnai 10 TEM (FEI). Images were captured with a Megaview G2 camera and processed with iTEM and Adobe Photoshop software.

### Protein Extraction of MVs and EXOs

MVs and EXOs pellet were lysed in cold Radio-Immunoprecipitation Assay (RIPA) buffer containing a mixture of phosphatase and protease inhibitors (Sigma-Aldrich, Italy). They were incubated for 20 min in ice and centrifuged at 16,000 g for 5 min at 4°C. The supernatant was transferred to a fresh tube and protein concentration was determined by BCA assay (Sigma-Aldrich, Italy). The insoluble fraction was processed as described in FRA section.

### Western Blot Analysis

An amount of 30 μg of MVs and EXOs lysates were loaded on SDS 12.5% polyacrylamide gels with a Mini-PROTEAN^®^ Tetra Vertical Electrophoresis Cell (BioRad, Italy) transferred to a nitrocellulose membrane (BioRad, Italy), using a semi-dry transfer apparatus (Trans-blot, BioRad, Italy) and blocked with 5% non-fat dry milk in Tween-20 Tris-Buffered Saline solution (TBS-T) (blocking solution) for 1 h. Membranes were incubated overnight with a mouse monoclonal primary antibody anti-TDP-43 (Proteintech, Inc., United States), which recognizes the full-length protein as well as all post-translationally modified (phosphorylated and glycosylated forms) and truncated forms in multiple applications in blocking solution. Membranes were then incubated for 1 h at room temperature with donkey anti-mouse secondary peroxidase-conjugated antibody (GE Healthcare, United Kingdom). Bands were visualized using an enhanced chemiluminescence detection kit (ECL Advance, Ge Healthcare, United Kingdom). For subsequent immunoreactions, primary and secondary antibodies were removed from the membrane with stripping solution (100 mM Glycine, 0.1% NP-40, 1% SDS pH 2.2) incubated for 20 min. Membranes were then washed with TBST and processed, as previously described with SOD1-DSE2-3H1 antibody (kindly given by Prof. Neil R. Cashman), SOD1 antibody (Santa Cruz Biotechnology, United States), which recognizes all SOD1 independently from its subtype, and FUS antibody (GeneTex, United States). To confirm the purity of MVs and EXOs preparations, anti-Annexin V (Santa Cruz Biotechnology, Inc., United States) or Anti-Alix (Abcam, Inc., United States) antibodies were used, respectively. Densitometric analysis of the bands was performed using ImageJ software (National Institutes of Health, United States). Densitometric assessment in arbitrary units was carried out in each EVs fraction.

### Filter Retardation Assay (FRA)

Twelve micrograms of protein extracts (quantified used the BCA assay as described above) were prepared in a volume of 100 μL of RIPA buffer and loaded onto 0.2 μm cellulose acetate membrane (Whatman, GE Healthcare, United Kingdom) and filtered through a Bio-Dot SF Microfiltration Apparatus (BioRad, Italy). Slot-blots were probed as described for WB to detect retained SOD1 insoluble species. Densitometric optical analysis of slot-blots and their relative ponceau (used for loading control) was performed and represented as mean ± SEM.

### Statistical Analysis

Results of independent experiments were expressed as mean ± SEM. Statistical tests were performed using a GraphPad Prism program. T test and one-way ANOVA with Bonferroni’s multiple comparison test was performed. A *p* < 0.05 was considered statistically significant.

## Results

### Microvesicles and Exosomes Sizes Are Enhanced in Plasma of ALS Patients

**FIGURE 2:**
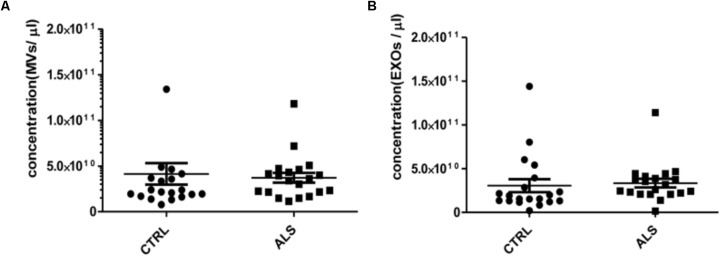
MVs and EXOs abundance do not differ between ALS patients and controls. NTA was performed on MVs and EXOs extracted from plasma of healthy controls and ALS patients (*n* = 20 per group). When averaged, the concentration of MVs **(A)** and EXOs **(B)** was not significantly different between the two groups (for MVs CTRLs 4.14 ×10^10^± 1.18 × 10^10^ vs. ALS 3.72 × 10^10^ ± 5.44 × 10^9^ particles/mL and for EXOs CTRLs 3.05 ×10^10^± 7.40 × 10^9^ vs. ALS 3.35 × 10^10^ ± 4.96 × 10^9^ particles/mL).

**FIGURE 3:**
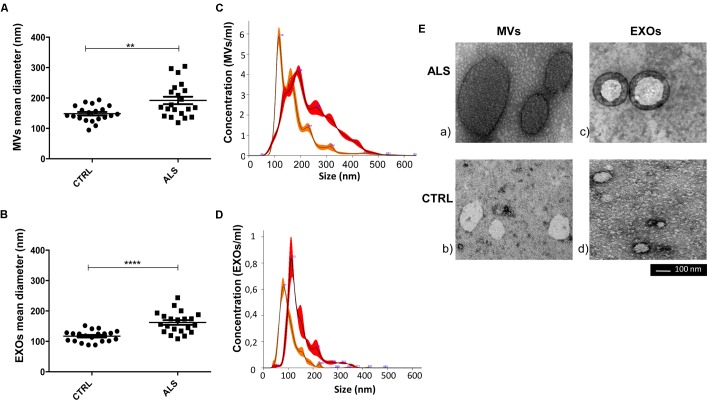
Mean diameter of MVs and of EXOs from ALS patients were significantly higher than healthy donors (CTRLs). Dot plot of the mean size of plasma derived MVs **(A)** and EXOs **(B)** of 20 ALS patients and 20 healthy individuals are shown (*t*-test, ^∗∗^*p* < 0.01, ^∗∗∗∗^*p* < 0.0001). **(C,D)** NTA profiles overlay between ALS MVs (red line-mean = 234.3 ± 1.7 nm) and CTRL MVs (orange line-mean = 174.7 ± 1.8 nm) and ALS EXOs (red line-mean = 150.3 ± 3.5 nm) and CTRL EXOs (orange line-mean = 103.7 ± 0.7 nm). The peak size represents the mode; **(E)** Representative images obtained by transmission electron microscopy (TEM) of MVs and EXOs of an ALS patient (ALS) and a healthy control (Scale bar: 100 nm). Images were cropped and original images are shown in Supplementary Figure 1. Diameter of MVs and of EXOs from ALS patients were significantly higher than healthy donors (Supplementary Figures 1A,B for MVs; Supplementary Figures 1C,D for EXOs). Full figures are represented in Supplementary Figure 1.

MVs and EXOs were purified from plasma of 20 healthy donors by differential centrifugation and filtration protocol (Thery et al., 2006), and NTA was used to identify their dimension and concentration. As shown in **Figure 1A**, the mode size of EXOs and MVs was 96.7 nm ± SD 4.7 nm and 142.8 nm ± SD 6.0 nm, respectively. Different size of MVs and of EXOs were also confirmed by TEM (**Figure 1B**). Plasma derived MVs and EXOs from 20 healthy controls showed the same concentration (4.14 × 10^10^ particles/mL ± 1.18 × 10^10^ particles/ml for MVs and 3.05 × 10^10^ particles/mL ± 7.4 × 10^9^ particles/mL) (**Figure 1C**). According to the literature, purity of MVs and EXOs was tested by detection of classical MVs and EXOs enriched markers by WB (Hugel et al., 2005; Baietti et al., 2012; Lötvall et al., 2014). As shown in **Figure 1D**, MVs were recognized by Annexin V and Integrin α2β, whilst EXOs were enriched in Alix and Flotillin 1. Once confirmed the purity of MVs and EXOs pellets, we also performed NTA (Nanoparticle Tracking Analysis) on MVs and EXOs derived from plasma of ALS patients to check the difference in concentrations and sizes compared to controls. MVs and EXOs concentrations did not differ between ALS patients and healthy controls (for MVs CTRLs 4.14 × 10^10^ ± 1.18 × 10^10^ vs. ALS 3.72 × 10^10^ ± 5.44 × 10^9^ particles/mL and for EXOs CTRLs 3.05 × 10^10^± 7.40 × 10^9^ vs. ALS 3.35 × 10^10^ ± 4.96 × 10^9^ particles/mL) (**Figures 2A,B**). Surprisingly, mean dimension resulted increased in ALS patients compared to controls, both for MVs (CTRLs 148.4 nm ± 5.818 nm vs. ALS 192.0 nm ± 12.13 nm, ^∗∗^*p* < 0.01) (**Figure 3A**) and for EXOs (CTRL 117.0 nm ± SD 4.127 nm vs. ALS 162.1 nm ± SD 7.604 nm, ^∗∗∗^*p* < 0.001) (**Figure 3B**). On average, MVs and EXOs derived from plasma of ALS patients were about 40 nm bigger than the CTRLs in both cases. The difference in size between healthy donors and ALS MVs and EXOs distribution is also clearly shown in **Figures 3C,D**. These data were confirmed by TEM (**Figure 3E**).

### MVs and EXOs From ALS Patients Are Enriched With SOD-1, TDP-43, and FUS Proteins

**FIGURE 4:**
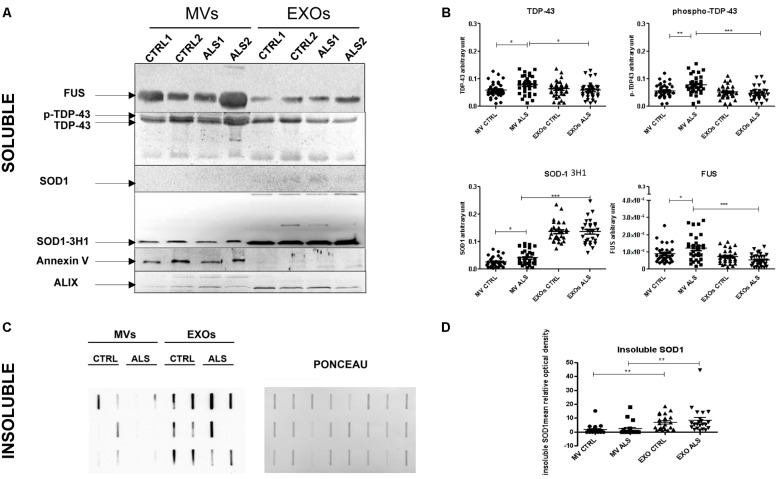
Plasma derived MVs from ALS patients are enriched with pathological proteins compared to controls. **(A)** SOD1, TDP-43, and FUS detection by Western Blot analysis in the soluble fraction of MVs and EXOs derived from plasma of 2 ALS and 2 healthy donors. Annexin V and Alix were used as marker control for MVs and EXOs. SOD1, recognized by specific antibody 3H1 (but not by polyclonal antibody anti-SOD1) was present in MVs and EXOs of ALS patients compared to healthy controls. TDP-43 bands and FUS were recognized in MVs and in EXOs. Images are cropped for clarity and full figures are shown in Supplementary Figures 2–4; **(B)** TDP-43, p-TDP-43, SOD1, and FUS densitometric analysis in MVs and EXOs soluble fraction from 30 ALS patients and matched controls. (ANOVA test, ^∗^*p* < 0.05, ^∗∗^*p* < 0.01, ^∗∗∗^*P* < 0.001). ALS patients have an increased level of SOD1, TDP-43, FUS level in MVs compared to controls. No variation was observed in EXOs of patients and controls; **(C)** SOD1 insoluble fraction in MVs and EXOs from 6 CTRLs and 6 ALS patients. Example of filter retardation assay probed with polyclonal anti-SOD1 antibody (Santa Cruz Biotechnology, United States) and its Ponceau; **(D)** SOD1 densitometric analysis in MVs and EXOs insoluble fraction from 22 ALS patients and matched controls. (ANOVA test, ^∗∗^*P* < 0.01). SOD1 protein level was not different between ALS and CTRLs group neither in MVs and EXOs but the real difference was between EXOs and MVs, since the latter have less protein than the first.

We investigated if ALS pathological proteins such as SOD1, TDP-43, and FUS in EVs differ between EVs from plasma of ALS patients compared to age- and sex-matched healthy controls (each group was composed of 30 individuals). Annexin V and Alix were used as common markers for MVs and EXOs fraction. We used two antibodies to detect SOD1: a monoclonal one (SOD1-DSE2-3H1) and a polyclonal antibody (SOD1). SOD1 (evaluated using the specific DSE2-3H1 antibody) was detected in both EVs types and in both ALS and healthy CTRL SOD1. Overall SOD1 was more concentrated in EXOs than in MVs (*p* < 0.001). In EXOs we did not find differences in SOD1 concentration between ALS patients and healthy CTRL; while, in MVs derived from ALS patients SOD1 protein levels were higher than CTRLs (*p* < 0.05) (**Figure 4B**). The polyclonal antibody against SOD1 did not recognize any protein in the soluble fraction of MVs and EXOs from plasma of either ALS or healthy controls (**Figure 4A**). However, by analyzing the aggregated SOD1 using filter retardation assay (FRA), we found SOD1 in the insoluble fraction (**Figure 4C**). SOD1 reactive species, retained on the cellulose acetate membrane, were similar in ALS and CTRL groups either in MVs or in EXOs, even though EXOs contained more insoluble SOD1 species than MVs (**Figure 4D**). The monoclonal antibody against TDP-43 recognized two bands at 43 and 45 KDa, the intact protein and its phosphorylated isoform. Interestingly, TDP-43 and its phosphorylated (p-TDP-43) form were slightly higher in MVs than EXOs derived from ALS patients (*p* < 0.001). Further, TDP-43 and mostly p-TDP-43 levels were statistically higher in MVs derived from ALS patients than from CTRLs (*p* < 0.05, *p* < 0.001) (**Figure 4B**). FUS protein followed the trend observed for p-TDP-43. In fact, FUS protein levels were slightly higher in MVs than in EXOs (*p* < 0.001) and differentially concentrated in MVs of ALS patients compared to controls (*p* < 0.05).

## Discussion

Microvesicles (MVs) and exosomes (EXOs) are EVs of distinct dimension and biological functions. This study is the first one that evaluated the dimension, the concentration and ALS-related protein cargo of MVs and EXOs from plasma of ALS patients compared to healthy controls. Plasma derived MVs and EXOs were separated by differential centrifugation and filtration. This method led to the isolation of MVs and EXOs with an overlap of dimension as described in the literature (Gardiner et al., 2016). However, evidence from imaging, NTA and western blotting indicated that our protocol separated and effectively minimized contamination of MVs in EXOs and *viceversa*. Plasma derived MVs and EXOs from ALS patients were significantly bigger than healthy controls. In ALS EVs were described to be involved in the release and uptake of pathological proteins only in cell cultures, but not in human biofluids (Gomes et al., 2007; Urushitani et al., 2008; Munch and Bertolotti, 2011; Basso et al., 2013; Nonaka et al., 2013; Feneberg et al., 2014; Grad et al., 2014; Iguchi et al., 2016; Pinto et al., 2017).

Previous studies reported that EVs derived from cultured PC12 cells had a larger average size in electron microscopy (EM) images and this was due to the greater numbers of vesicular transporters or neurotransmitters (Colliver et al., 2000; Daniels et al., 2006). Significant increased diameter suggests that ALS EVs are enriched by macromolecules in response to the disease process (Needham and Nunn, 1990). In fact, we found a significant enrichment of SOD1, TDP-43, p-TDP-43, and FUS in MVs of ALS patients compared to CTRLs. While in MVs the enhancement of protein cargo can justify the enhancement in size, this cannot be said for EXOs, which do not have the same protein variation compared to healthy control. Probably, in physiological conditions EXOs are the principal way to scavenge unfolded proteins, while during the disease when the bulk of the pathological proteins is largely increased, the MVs system is activated. We found an enhanced level of pathological proteins in plasma derived MVs from ALS patients compared to controls. The enhanced size of EXOs could be caused by a selective enrichment with other biomolecules such as lipids, other proteins and RNAs (Raposo and Stoorvogel, 2013). Studies of their cargo could confirm the different regulation of the two classes of EVs and their selectivity in the transport of macromolecules.

In addition, we found that SOD1 was generally enriched in EXOs compared to MVs, while p-TDP-43 and FUS were slightly more concentrated in MVs than in EXOs. This result underlines a selectivity of MVs and EXOs in protein cargo transport. mutSOD1 is consistently associated with JUNQ (juxtanuclear quality control compartment)-like inclusions, microtubule dependent inclusions enriched in ubiquitilated proteins, proteasome subunits and chaperones such as Hsp70 (Kaganovich et al., 2008). Maturation of late endosomes is governed by microtubules movement toward the cell center in a dynein-dependent fashion, so this might explain SOD1 enrichment in EXOs compared to MVs (Martin-Cofreces et al., 2014). Farrawell et al. demonstrated that TDP-43 and FUS can form JUNQ-like inclusions, but also microtubule-independent inclusions, called insoluble protein deposit (IPOD) compartment. These inclusions (differently from SOD1 inclusions) can be formed slowly and can incorporate large complexes, like mRNA or DNA, and they might migrate in stress granule (SGs) formations (Farrawell et al., 2015). Removal of SGs is also regulated by autophagy pathways (Buchan et al., 2013) and insufficient digestion of damaged molecules might promote an alternative release of these molecules through EXOs. On the other hand, MVs production can be significantly elevated under various stressor stimulations, including increased concentration of Ca^2+^, cellular stress and immune system response (Akers et al., 2013), events that can be caused by SOD1, TDP-43, and FUS aggregation in ALS. It was observed that SOD1 aggregation can trigger Ca^2+^ overload (Tradewell et al., 2011) and in turn, that Ca^2+^ can bind to SOD1 promoting its aggregation (Estaìcio et al., 2015). Deregulated Ca^2+^ levels can activate calpain protease that cleaves TDP-43 at the C-terminal, generating aggregation prone N-terminal segments that are found in the majority of ALS patients (Aggad et al., 2014; Yamashita and Kwak, 2014). Mutation of FUS protein leads to CAMK2N2 up-regulation (Convertini et al., 2013), which phosphorylates AMPA receptors inducing Ca^2+^ release. In summary, the literature can partially explain how SOD1, TDP-43, and FUS can be transported in MVs under pathological stresses, however, further studies are needed.

To underline the impact of MVs on ALS diseases, we have already demonstrated that leukocyte derived MVs (LMVs) are able to transport pathological proteins like p-TDP-43 and in particular SOD1. The regulation of LMVs formation might exert a neuroprotective effect by SOD1 protein sequestration, which have a significative impact on disease progression (Sproviero et al., unpublished). In this study we demonstrated that MVs of ALS patients are enriched with potentially pathological (SOD1, TDP-43, p-TDP-43, FUS), compared to CTRLs while EXOs do not show any protein changes, even if both types of EVs have bigger size compared to controls. These two classes of EVs are complex systems of scavengers and further studies are needed to understand if lipids or RNA might enhance their size. However, according to our previous study, these data postulate that MVs can act as scavangers of toxic proteins, which are known to be involved in prion-like distribution in ALS disease.

## Permission to Reuse and Copyright

The use, distribution or reproduction in other forums is permitted and the original publication in this journal is cited, in accordance with accepted academic practice. No use, distribution or reproduction is permitted which does not comply with these terms. Permission must be obtained for use of copyrighted material from other sources (including the web). Please note that it is compulsory to follow figure instructions.

## Data Availability Statement

All data for this study are included in the manuscript and the Supplementary Files.

## Ethics Statement

This study was carried out in accordance with the recommendations of Protocol n. 375/04 – version 07/01/2004, Ethical Committee of the National Neurological Institute “C. Mondino,” IRCCS (Pavia, Italy). The protocol was approved by Ethical Committee of the National Neurological Institute “C. Mondino,” IRCCS (Pavia, Italy). All subjects gave written informed consent in accordance with the Declaration of Helsinki.

## Author Contributions

DS experimented execution, designed the study, acquired and analyzed the data, and drafted the manuscript and figures. SLS experimented execution, designed the study, and acquired and analyzed the data. MG acquired and analyzed the data. VC experimented execution, acquired and analyzed the data, and revised the manuscript. SG and OP designed the study, acquired and analyzed the data, and revised the manuscript. AP revised the manuscript. SB recruited controls individuals. LD and MC recruited ALS patients. CC conceived, designed the study, and revised the data analysis and the manuscript.

## Conflict of Interest Statement

The authors declare that the research was conducted in the absence of any commercial or financial relationships that could be construed as a potential conflict of interest.

## Abbreviations

ALS: amyotrophic lateral sclerosis
EVs: extracellular vesicles
EXOs: exosomes
MVs: microvesicles
SGs: Stress Granules
