# Supplementary material for: Pathological Proteins Are Transported by Extracellular Vesicles of Sporadic Amyotrophic Lateral Sclerosis Patients
Source: Front Neurosci. 2018 Jul 19;12:487. doi: 10.3389/fnins.2018.00487 (PMC6060258; doi:10.3389/fnins.2018.00487)

Supplementary Material

Pathological proteins are transported by extracellular vesicles of sporadic Amyotrophic Lateral Sclerosis patients

Daisy Sproviero[1], Sabrina La Salvia[1], Marta Giannini[1,2], Valeria Crippa[3], Stella Gagliardi[1], Stefano Bernuzzi[4], Luca Diamanti[2,5], Mauro Ceroni[2,5], Orietta Pansarasa[1], Angelo Poletti[3], Cristina Cereda[1*].

*** Correspondence:** Dr. Cristina Cereda, e-mail: cristina.cereda@mondino.it

Supplementary Figure 1. Transmission electron microscopy (TEM) of MVs and EXOs of an ALS patient and a healthy control. Diameter of MVs and of EXOs from ALS patients were significantly higher than healthy donors (A and B for MVs; C and D for EXOs). Exosomes were negatively stained with 2% uracyl acetate.

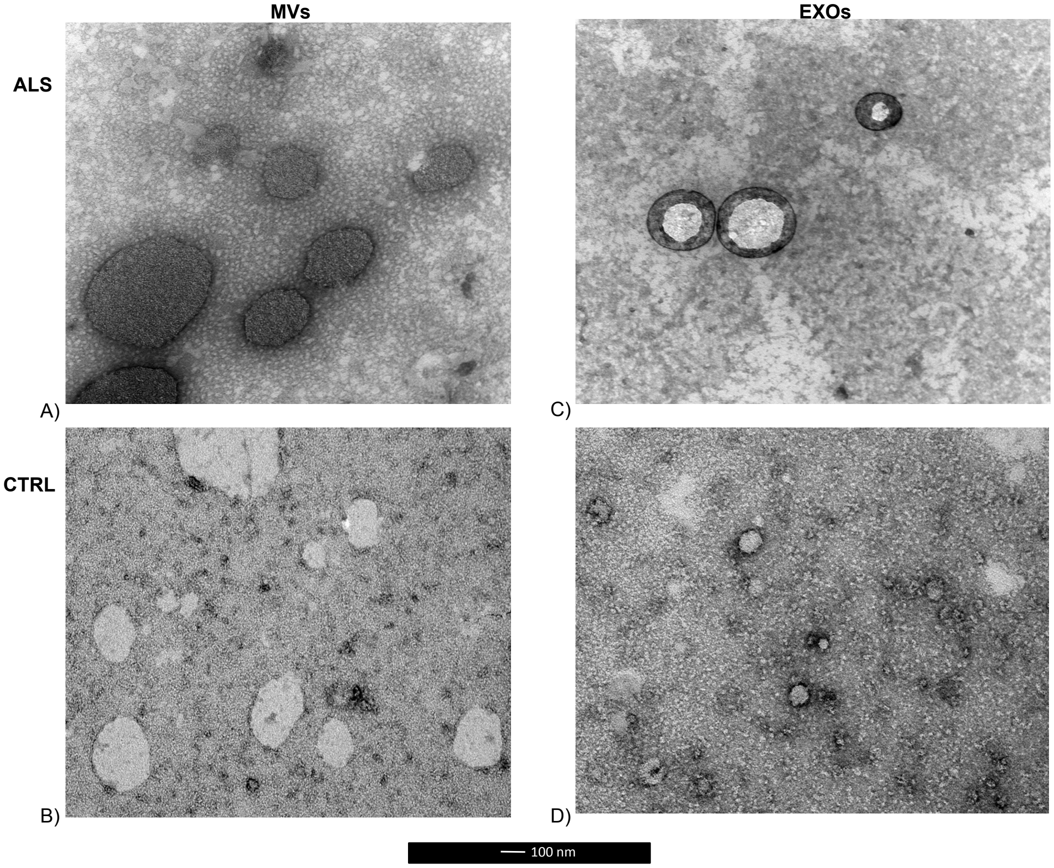

Supplementary Figure 2. Immunoblots of MVs and EXOs (30 µg protein) from plasma of two healthy donors (CTRL1 and CTRL2-MVs-lane 1, 2; CTRL1 and CTRL2-EXOs-lane 5, 6) and two ALS patients (ALS1 and ALS2-MVs-lane 3, 4; ALS1 and ALS2-EXOs-lane 7, 8) with anti-TDP-43 and p-TDP-43 (A) and anti-SOD1(B). Black squares show the cropping locations.

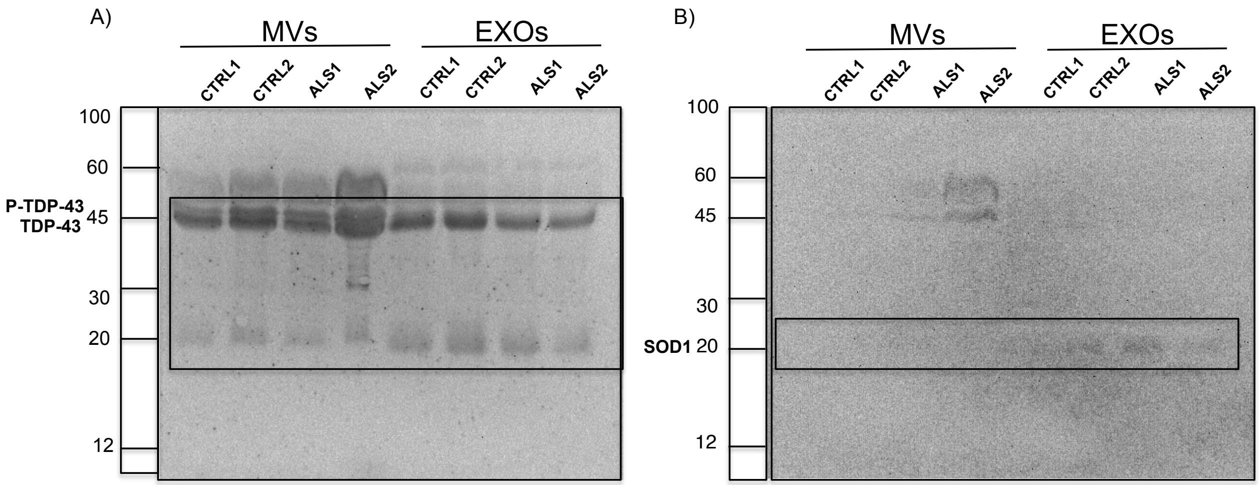

Supplementary Figure 3. Immunoblots of MVs and EXOs (30 µg protein) from plasma of two healthy donors (CTRL1 and CTRL2-MVs-lane 1, 2; CTRL1 and CTRL2-EXOs-lane 5, 6) and two ALS patients (ALS1 and ALS2-MVs-lane 3, 4; ALS1 and ALS2-EXOs-lane 7, 8) with anti-SOD1-3H1 (A) and anti-FUS (B). Black squares show the cropping locations.

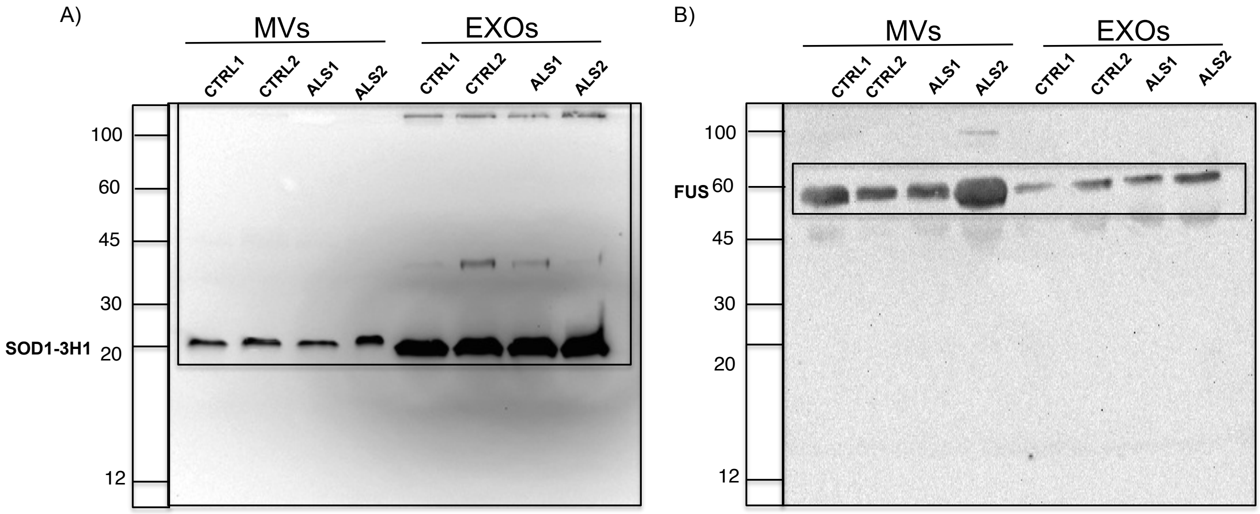

Supplementary Figure 4. Immunoblots of MVs and EXOs (30 μg protein) from plasma of two healthy donors (CTRL1 and CTRL2-MVs-lane 1, 2; CTRL1 and CTRL2-EXOs-lane 5, 6) and two ALS patients (ALS1 and ALS2-MVs-lane 3, 4; ALS1 and ALS2-EXOs-lane 7, 8) with anti-Annexin V (A) and anti-Alix (B). Black squares show the cropping locations.

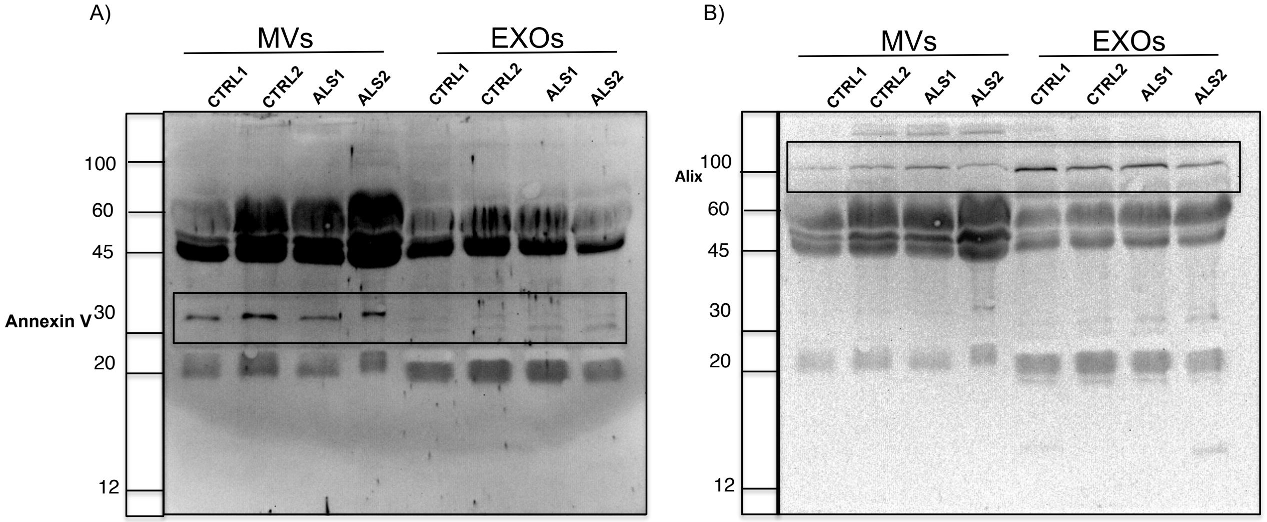

Supplement: Supplementary file 1 [file Image_1.pdf]
